# 17β-estradiol ameliorates delirium-like phenotypes in a murine model of urinary tract infection

**DOI:** 10.1038/s41598-022-24247-w

**Published:** 2022-11-15

**Authors:** Gena Guidry, Nicklaus A. Sparrow, Hyyat S. Marshall, Roberta De Souza Santos, Suman P. Bharath, Michael M. Gezalian, Margareta D. Pisarska, Jean-Philippe Vit, Scott A. Kelly, S. Ananth Karumanchi, Shouri Lahiri

**Affiliations:** 1 Department of Neurology, Cedars-Sinai Medical Center, 8700 Beverly Blvd., Los Angeles, CA 90048 USA; 2 Department of Medicine, Cedars-Sinai Medical Center, Los Angeles, CA USA; 3 Department of Neurosurgery, Cedars-Sinai Medical Center, Los Angeles, CA USA; 4 Division of Reproductive Endocrinology and Infertility, Department of Obstetrics and Gynecology, Cedars-Sinai Medical Center, Los Angeles, CA USA; 5 David Geffen School of Medicine, University of California, Los Angeles, CA USA; 6 Department of Biomedical Sciences, Cedars-Sinai Medical Center, Los Angeles, CA USA

**Keywords:** Urinary tract infection, Neurology, Neuroscience

## Abstract

Urinary tract infections (UTIs) are common and frequently precipitate delirium-like states. Advanced age coincident with the postmenopausal period is a risk factor for delirium following UTIs. We previously demonstrated a pathological role for interleukin-6 (IL-6) in mediating delirium-like phenotypes in a murine model of UTI. Estrogen has been implicated in reducing peripheral IL-6 expression, but it is unknown whether the increased susceptibility of postmenopausal females to developing delirium concomitant with UTIs reflects diminished effects of circulating estrogen. Here, we tested this hypothesis in a mouse model of UTI. Female C57BL/6J mice were oophorectomized, UTIs induced by transurethral inoculation of *E. coli*, and treated with 17β-estradiol. Delirium-like behaviors were evaluated prior to and following UTI and 17β-estradiol treatment. Compared to controls, mice treated with 17β-estradiol had less neuronal injury, improved delirium-like behaviors, and less plasma and frontal cortex IL-6. In vitro studies further showed that 17β-estradiol may also directly mediate neuronal protection, suggesting pleiotropic mechanisms of 17β-estradiol-mediated neuroprotection. In summary, we demonstrate a beneficial role for 17β-estradiol in ameliorating acute UTI-induced structural and functional delirium-like phenotypes. These findings provide pre-clinical justification for 17β-estradiol as a therapeutic target to ameliorate delirium following UTI.

## Introduction

Urinary tract infections (UTIs) affect millions of individuals each year and frequently induce delirium-like states^1,2^. Delirium-like states can broadly be defined as acute confusion with behavioral impairments including, inattentiveness, short-term memory impairment, and psychomotor agitation^3–5^. UTI concomitant with delirium is known to contribute to increased mortality, prolonged hospitalizations, and long-term cognitive impairment, particularly in those with pre-existing neurodegenerative pathologies^6–8^. Advanced age coinciding with the postmenopausal period and lower levels of estrogen is a well-established risk factor for delirium in women who are significantly more susceptible to developing recurrent UTIs^9–12^.

We previously published findings demonstrating a pathological role for interleukin-6 (IL-6) in mediating delirium-like phenotypes in response to UTI^13^. Specifically, we demonstrated that compared to non-UTI controls, mice with UTIs have significantly higher plasma IL-6, greater impairments in frontal and hippocampus-mediated behaviors, and elevated neuronal cleaved caspase-3 (CC3), a known early marker of apoptosis^14–17^. We further showed that treatment with systemic anti-IL-6 reversed these functional and structural impairments including the UTI-induced increases in frontal/hippocampal CC3. Since neurons do not express the IL-6 receptor, we hypothesized that the systemic IL-6/soluble IL-6 receptor (sIL-6R) complex may induce delirium-like phenotypes via the IL-6 *trans*-signaling pathway as proposed in diverse neurodegenerative pathologies^18,19^. In the *trans*-signaling pathway, IL-6 binds to sIL-6R in the systemic circulation and directly elicits signaling in distal endothelial or neuronal cells via engagement of the glycoprotein 130 receptor, without needing to bind to membrane bound signaling receptors, ultimately culminating in neuronal injury and neurodegeneration via upregulation of CC3^20,21^. In contrast, in the classical signaling pathway, IL-6 binds to membrane-bound IL-6R expressed in a limited variety of cells such as microglia, leading to activation of anti-inflammatory signals. However, in pathological states, microglia can switch from neuroprotective to activated neurotoxic phenotypes that produce pro-inflammatory IL-6/sIL-6R and contribute to neuronal injury via positive amplification of the IL-6 *trans*-signaling pathway.

While prior studies have demonstrated the role of estrogen in reducing peripheral IL-6 expression^22,23^, it remains unknown whether the increased susceptibility of postmenopausal females to developing delirium with UTI reflects diminished effects of circulating estrogen on suppressing peripheral IL-6. Accordingly, in this study, we sought to test the hypothesis that peripheral 17β-estradiol ameliorates acute delirium-like phenotypes via modulation of peripheral IL-6 using a murine model of UTI.

## Methods

### Animals

**Figure 1:**
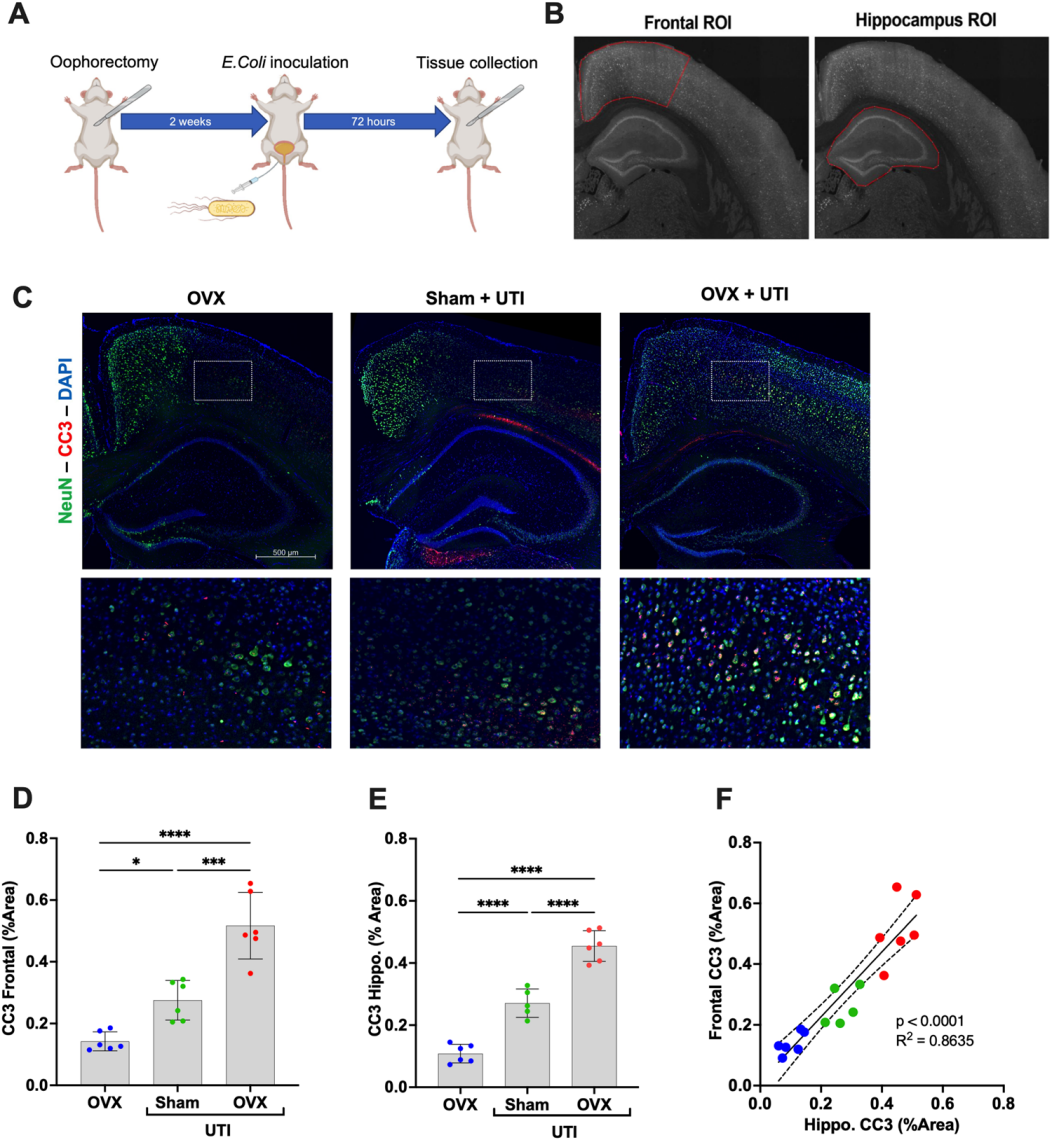
Oophorectomy (OVX) and urinary tract infection (UTI)-mediated changes in cleaved caspase-3 (CC3). (**A**) A schematic of design of a preliminary experiment examining the effects of OVX, UTI, and OVX + UTI on levels of cortical and hippocampal CC3. (**B**) Representative regions of interest (ROIs) in the frontal cortex and hippocampus. (**C**) Representative staining of CC3 and neuronal nuclear protein (NeuN) in each group (**D**,**E**) Quantification of CC3 in the frontal cortex and hippocampus demonstrates a statistically significant increase in CC3 in mice with UTIs alone [sham (green), n = 6] compared to mice with OVX alone (blue, n = 6). Additionally, among mice with UTIs, OVX animals (red, n = 6) had significantly higher levels of CC3 compared to the sham group (green). (**F**) Regression analysis across groups demonstrates a statistically significant relationship between frontal cortex and hippocampal CC3 levels. Data are expressed as mean ± SD. Dotted lines represent 95% confidence intervals. *p < 0.05, ***p < 0.001, ****p < 0.0001.

**Figure 2:**
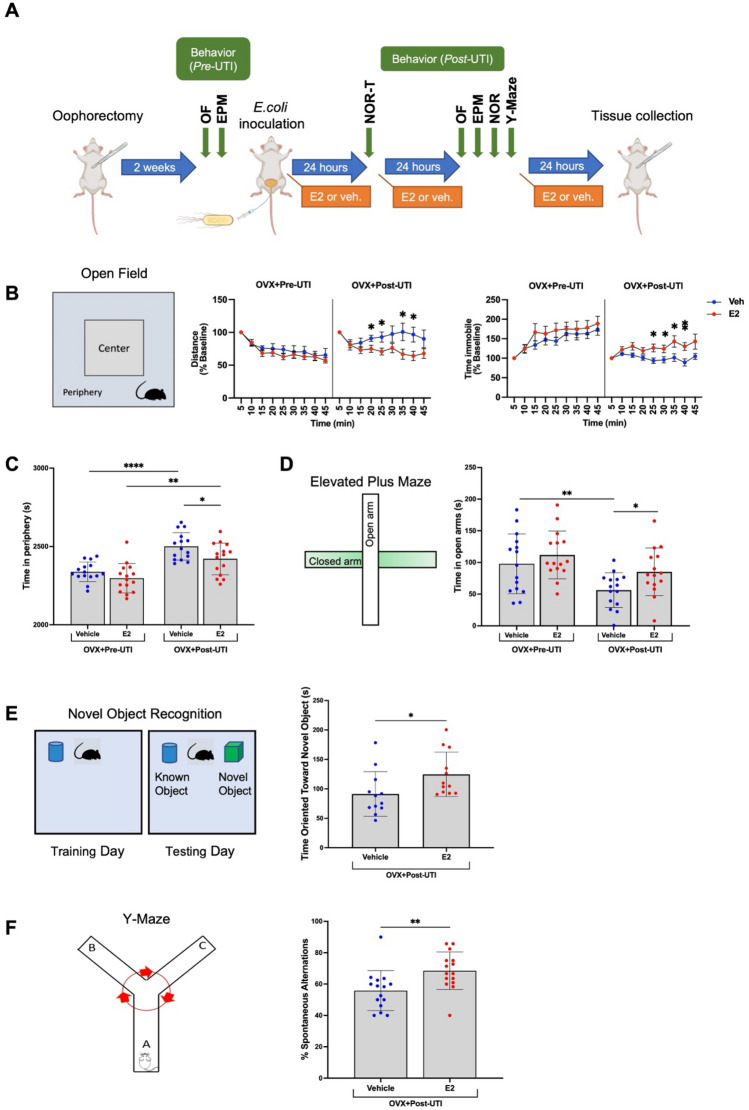
17β-estradiol (E2) significantly ameliorates UTI-induced delirium-like behavior. (**A**) Schematic of primary experimental design and treatment schedule of E2. Note that to prevent any habituation, novel object recognition (NOR) and Y-maze behavioral tests were only utilized *post* UTI induction and E2 (or vehicle) treatment. At a given time point behavioral tests were performed sequentially. NOR-T represents training the training phase of the behavioral test. (**B**) Distance (m) and time immobile (sec) as a percent of baseline (initial 5 min). Prior to UTI induction and E2 treatment (OVX + Pre-UTI), there were no treatment effects but a statistically significant effect of time over the course of the 45-min open field test (OF); decreased distance travelled (*F*_3,110_ = 22.2; p < 0.0001) and increased time spent immobile (*F*_5,171_ = 14.8; p < 0.0001). Following UTI (OVX + Post-UTI) there was statistically significant time-by-E2 treatment interactions for distance traveled (*F*_24,496_ = 1.9; *p* = 0.0052) and time spent immobile (*F*_24,448_ = 3.0; p < 0.0001). Results of *post*-hoc tests, comparing treatment effects (vehicle vs. E2) at individual time points, are indicated by asterixis. (**C**) Among oophorectomized and UTI inoculated mice (OVX + Post-UTI), mice treated with E2 spent statistically significantly less time in the periphery of the open field. (**D**–**F**) Mice (OVX + Post-UTI) treated with E2 spent statistically significantly more time in the open arm (elevated plus maze, EPM), more time and oriented towards a novel object (novel object recognition), and more % spontaneous alterations (Y-maze) compared to the vehicle treated group. Following oophorectomy, but prior to UTI induction and E2 treatment (OVX + Pre-UTI), there were no statistically significant behavioral differences between groups. Group size was *n* = 15. Data are expressed as mean ± SD. *p < 0.05, **p < 0.01, ****p < 0.0001.

An initial experiment was performed using 18 females C57BL/6J mice, aged 6–7 months, that had not previously had litters (Jackson Laboratory, Bar Harbor, ME). Mice were randomized into three groups: oophorectomized (OVX, n = 6), UTI inoculated (Sham, n = 6), and oophorectomized and UTI inoculated (n = 6). For the associated timeline see Fig. 1A. In the primary experiment (see Fig. 2A for experimental design schematic), a total of 48 female C57BL/6J mice, aged 6–7 months (no prior litters) (Jackson Laboratory, Bar Harbor, ME), were utilized. Mice were randomized to one of the following groups: oophorectomized (OVX), UTI inoculated, and treated with 7β-estradiol (n = 24); or OVX, UTI inoculated and treated with a vehicle (sesame oil, n = 24). We utilized a total of 4 cohorts (n = 6/group/cohort, 24 total mice/group) to restrict the time of day at which behavioral assessments were performed—thus, minimizing any variability due to natural circadian variations. In each of 4 cohorts, treatment groups were equally represented, and mice were randomized across the two groups. In each cohort methods were identical, including close approximations of time of day of each procedure. All dependent variables were measured in cohorts 1–3 with the following exception: novel object recognition test was measured in cohorts 3 and 4 only. CC3 was evaluated in all 4 cohorts. Data were analyzed and displayed combining all 4 cohorts where applicable. All mice were housed in Cedars-Sinai’s AAALAC accredited animal facility under standard conditions (kept in ventilated cages at approximately 21 °C, 40–70% humidity, a 12-h light/dark cycle, with food and water available to the animals ad libitum). All procedures herein follow the recommendations in the ARRIVE guidelines for research involving the use of animals. For testing procedures, order was randomized within a day to control for potential confounders.

### UTI model and 17β-estradiol treatment

We have previously published the following methods (including all following subsections) and the original source of the method descriptions can be found elsewhere^13^. Here, we replicate the methods and provided details of any modifications and/or additions. Static cultures of *E. coli* (Migula) Castellani and Chalmers (ATCC^®^ 700928™) strain (CFT073) were grown for 24 h at 37 °C. Bacterial cultures were harvested by centrifugation (4000 rpm for 10 min at 4 °C) and resuspended in sterile endotoxin-free phosphate-buffered saline (PBS; PromoCell, Heidelberg, Germany) at a concentration of 1 × 10^9^ colony forming unit/mL (CFU/mL). Mice were inoculated transurethrally as previously described^13,24^. Briefly, 6–7-month-old female C57BL/6J mice were quarantined for a minimum of 1 week prior to inoculation and allowed food and water ad libitum. On Day 0 (Fig. 2A), mice were anesthetized with isoflurane USP (Clipper Distribution Company LLC, St. Joseph, MO) delivered by a vaporizer (Vet Equip Inc., Pleasanton, CA) set at 2% isoflurane and 0.8 L of oxygen per minute. The inoculum was delivered via a 1-ml syringe connected to a 1/2-in., 30-gauge needle. The catheters were prepared with sterile 0.61-mm (internal diameter) polyethylene tubing (PE 10, Becton, Dickinson, Sparks, MD). The needle was inserted into the catheter, and 100 µl of inoculum containing 1 × 10^8^ CFU of *E. coli* was delivered. Urine samples were collected and processed bacterial enumeration and determination of the intensity of pyuria. Following inoculation with *E. coli* mice were treated with either vehicle (200 µl of sesame oil) or 17β-estradiol (7 µg in 200 µl of sesame oil; mean = 0.28 mg/kg, range 0.24–0.32 mg/kg) via intraperitoneal injection once a day for 3 days. The 17β-estradiol dose was selected based on previously observed protective effects on behavior and neuronal function in mouse studies^25,26^. After 3 days, mice were euthanized by perfusion, while deeply anesthetized with a combination of ketamine and dexmedetomidine, followed by a physical method for tissue and blood collection, and the brain was aseptically removed. Plasma collected at the time of tissue harvest was utilized for the quantification of IL-6 and IL-6R.

### Brain isolation and treatment

Mice were deeply anesthetized and perfused with room temperature PBS with 0.5 mM ethylenediaminetetraacetic acid (10 mL). Right hemispheres were collected and fixed by submerging in ice-cold PBS buffered 4% paraformaldehyde (Electron Microscopy Sciences) for 30 min, and then cryo-protected in 2% paraformaldehyde + 30% sucrose at 4 °C for 24–48 h. Free-floating, 30-μm-thick coronal brain cryosections were prepared and stored at 4 °C in PBS + 0.02% sodium azide until staining. Cortices were separated from the left hemispheres and were immediately frozen on dry ice. They were then stored at − 80 °C until protein extraction.

### Immunohistochemistry and microscopy

Sections were affixed to slides by air drying and subjected to heat-induced epitope retrieval for 10 min in antigen-retrieval solution (pH 6.0; Invitrogen) before permeabilization/blocking in 5% BSA + 0.25% Triton X-100 in PBS for 1 h at room temperature. Sections were then incubated at 4 °C overnight with primary antibodies diluted in 1% BSA + 0.01% Triton X-100 in PBS (Ab Diluent). After washing, sections were incubated with a combination of the appropriate secondary antibody (Alexa Fluor Plus conjugated; Invitrogen) diluted to 4 µg/mL in Ab Diluent for 1 h at room temperature. After washing, sections were incubated in 0.05% Sudan black B in 70% ethanol for 10 min to reduce tissue autofluorescence. Sections were mounted using ProLong Glass with DAPI (Invitrogen, Carlsbad, CA, USA). Negative controls were processed using the same protocol with the omission of the primary antibody to assess non-specific labeling. A Carl Zeiss AxioImager Z.2 epi-fluorescence microscope equipped with standard filter sets/mercury arch lamp, an Apotome 2.0, and an Axiocam HRm camera controlled by Zen Blue Pro (version 2.3) software was used to acquire and process images. Images of damage marker (i.e., CC3) staining were acquired with a 10× objective (NA 0.3, Zeiss) as a 5 × 5 tiled image that encompassed the frontal cortex and hippocampus of each section. All acquisition and display settings are the same between groups and settings were optimized using the UTI group. All images within a figure are set to the same scale.

### Image analysis

Fiji (ImageJ v. 1.53c) software was used for image analysis and semi-quantitation. Three coronal sections containing the cortex and hippocampus were analyzed (one ventral, one mid, and one dorsal) per animal. For damage marker analysis, two different regions of interest (ROIs) were drawn on tiled images of sections: a ROI around the cortex (with an average area of 315 µm^2^), or a ROI encompassing the entire hippocampus. A threshold was set to exclude background pixels per ROI based on the pixel intensity histogram, and the number of positive pixels was measured and then expressed as percent area of the ROI (e.g., see Fig. 1B). For cytokine analysis, a single field z-stack projection was analyzed per section. Background pixels were excluded per field based on the pixel intensity histogram and the intensity of the remaining pixels was used to calculate percent area. Values for each protein from the triplicate sections were averaged to yield one number per animal. Analysis was performed by assessors blinded to group allocation.

### Behavioral testing

Locomotor activity, anxiety, and memory were evaluated by subjecting individual mice to a series of behavioral tests prior to and following UTI induction and dose 2 of 17β-estradiol treatment (or vehicle) (Fig. 2A). Note that, to prevent any habituation to the apparatus and lack of motivation that may occur during subsequent exposures, novel object recognition and Y-maze behavioral tests were only performed *post* UTI induction and 17β-estradiol (or vehicle, sesame oil) treatment. Collectively, these behavioral tests (Fig. 2) are thought to model delirium-like phenotypes observed in humans: psychomotor agitation, inattentiveness, and short-term memory impairment^3^. We have previously utilized a subset of these behavioral tests to model delirium-like phenotypes in a UTI mouse model^13^.

#### Open field

Broadly, the open field test measures locomotor activity and exploratory behavior. Delirium-relevant behavioral features that can be assessed with the open field test include altered level of consciousness, psychomotor agitation, or retardation^27^. In normal mice, locomotor activity decreases over time due to habituation to the testing environment. Failure to habituate to the testing environment indicates the presence of altered consciousness while increased time spent in the periphery as opposed to the center of the open field maze indicates increased anxiety-like behavior. Locomotor activity and anxiety-related emotional behaviors were evaluated by placing individual mice in the center of an opaque Plexiglas (40 × 40 × 40 cm) arena and allowed to explore for 45 min^27,28^. The open field was divided into a 20 × 20 cm central zone (center) and a surrounding border zone (periphery) during the analysis (Fig. 2B). Activity in the open field was recorded by a computer-operated camera system (Stoelting Digital USB 2.0 CMOS Camera). Total distance or locomotor activity (m), movement time (sec), time in the center zone (sec), and time in the periphery (sec) were collected using ANY-maze Video Tracking Software version 7.1 (Stoelting Co., Wood Dale, IL, USA). Average speed (m/s) and time immobile (sec) were calculated. Total distance (m) and total time immobile (sec) were analyzed as a percent of baseline activity. Baseline activity was defined as the distance traveled or time spent immobile during the first five minutes of the 45-min test.

#### Elevated plus maze

Anxiety related behavior and altered level of consciousness was assessed utilizing an elevated plus maze (EPM)^28–30^. The EPM crossed arms are approximately 30 cm above the floor during testing, with open (no walls) and closed arms (walled) crossed perpendicularly to each other (Fig. 2D). Mice with delirium spend more time in the closed arms of the maze—avoidance of the open arms reflects higher anxiety-like behavior. A video camera was placed above the apparatus and ANY-Maze Video Tracking Software 7 (Stoelting Co., Wood Dale, IL, USA) was used to record movements. Individual mice were placed in the center of the crossed arms facing an open arm and allowed to freely explore the entire maze for 5 min. The EPM was cleaned with 70% EtOH solution between animals to eliminate odor traces. Time spent in open and closed arms of the elevated plus maze was quantified and time spent in open arms (sec) was analyzed.

#### Novel object recognition

To test non-spatial memory performance we utilized a novel object recognition memory test (NOR)^31^. Prior to testing the arena was cleaned with 70% EtOH before use. The training phase for the known object exposure occurred 24 h prior to and following UTI induction. During the training phase, a single object (known) was presented. During the training phase if mice exhibited biases for regions of the arena or did not explore the objects presented, they were excluded from the testing portion utilizing the novel object. The novel object, or test phase exposure, occurred after the treatment of 17β-estradiol (or vehicle), 48 h after UTI induction. During the test phase, in addition to the known object, a novel object was also presented (Fig. 2E). In normal mice, there is typically a preference for the novel object as the known object has been previously investigated. In mice with delirium, or altered acute memory, less preference for the novel object is demonstrated. All data was collected and scored using the AnyMaze (Stoelting Co., Wood Dale, IL, USA) data acquisition system. During all phases mice were habituated to the open field arena for at least 30 min prior to object exposure to minimize fear and/or anxiety. All objects were fixed to the floor before all exposure phases. The known and novel objects were both placed in the arena with enough distance between the wall and each other so that mice could freely explore the objects from all angles. Mice were allowed to explore objects for 10 min total during all phases and were not disturbed during this period. Object explorations were counted once the mouse was oriented towards the object, the snout was within 2 cm of the object, and the midpoint of the animal’s body was beyond 2 cm from the object. During the test phase the objects were placed in the same position as the training phase. We assessed novel object recognition by examining the time oriented toward each object.

#### Y-maze

The Y-Maze test was used to assess attentional, cognitive, and short-term memory^28,32,33^*. *The Y-maze consists of three arms with an angle of 120 degrees between each arm (Fig. 2F). Mice typically prefer to investigate a new arm of the maze rather than returning to one that was previously visited, as shown by alternated exploration of the 3 arms. In mice with delirium, we expect fewer alterations into a new arm as a percent of the total visits. Tests were performed in the manner previously described in^13^. Briefly, after treatment of 17β-estradiol or vehicle, mice are placed in the maze and allowed to freely explore the three arms for 10 min. AnyMaze (Stoelting Co., Wood Dale, IL, USA) tracking software was used to analyze the number and the sequence of arm entries, and the percentage of spontaneous alternations was calculated. Alternations are defined as the consecutive visits of the three different arms without returning to an arm previously visited. The Y-maze was cleaned with 70% EtOH solution between each mouse to eliminate odor traces.

### Cortical neuron culture and treatment

Primary rat cortical neurons were obtained from ThermoFisher Scientific (Gibco) and grown according to the manufacture’s protocol. Briefly, a 24-well plate precoated with poly-d-lysine (100 µg/mL for a minimum of 1 h at room temperature) was seeded with 4 × 10^5^ live cells (measured by BioRad TC10 Cell Counter, trypan blue-positive cells excluded) per well in 2 mL of growth medium. Neurons were grown in an incubator set to 37 °C and 5% CO_2_ in complete Neurobasal Plus medium (Neurobasal™ Plus base medium with 2% B-27™ Plus supplement, 2 mM GlutaMax™, and 1 × Antibiotic–Antimycotic), and half volume medium exchanges occurred every 24 h for 7 days. On the 7th day, neurons were treated with one of following conditions: (1) vehicle (growth medium), (2) 10 ng/mL IL-6 and 40 ng/mL sIL-6R (both from PeproTech, Cat# 200-06 and Cat# 200-06RC respectively), (3) IL-6 & sIL-6R plus 5 ng/mL 17β-estradiol (Millipore Sigma, Cat# 3301). Treatment medium (2 mL) was first allowed to equilibrate to the incubator for 30 min prior to treatment. The growth medium was then completely replaced with the pre-equilibrated treatment medium, and the plate was then incubated for 1 h, after which time protein extraction occurred*.*

### Western blot

Tissue culture plates were placed on ice, culture media was removed, and wells were washed two times with ice-cold PBS. Wells were then extracted with 95uL of ice-cold RIPA buffer with inhibitors. Protein concentrations were measured with Pierce, BCA protein assay kit (Thermo Scientific, USA). Equal amounts of proteins (8 μg for primary culture extracts) were denatured (95 °C for 5 min in SDS-sample buffer), separated on a SDS 4–12% polyacrylamide gel, and then transferred to a nitrocellulose membrane (Invitrogen, Carlsbad, CA, USA). Blots were blocked for 1 h at room temperature with 5% (w/v) BSA in PBST (PBS + 0.01% TX-100). Then the membrane was incubated at 4 °C with specific primary antibodies overnight. Primary antibodies used are listed in Table S1. After 3 wash steps with PBST, the blot was then incubated with the corresponding horseradish peroxidase‐conjugated secondary antibody (1:30,000). The blot was developed using Pierce Super Signal West Pico (Thermo Scientific, USA). Western blot images were acquired by iBright Western Blot Imaging Systems (Invitrogen, Carlsbad, CA, USA) and analyzed by iBright Analysis Software Version 3.1.3.

### Statistical analysis

As previously described^13^, based on preliminary analysis using CC3, a power analysis with one-way ANOVA and Tukey’s post-hoc test yielded greater than 95% power at the 0.05 significance level for an effect size of 1.1317 with a minimum of n = 8/group.

Prism 9.4.1 (GraphPad, https://www.graphpad.com/) was used for statistical analyses and analyses were performed by assessors blinded to group allocation. For the primary experiment (Fig. 2A), differences between experimental groups were evaluated using independent sample t-tests, while within group differences across time points (*pre* and *post* UTI) were evaluated using paired sample t-tests. For some open field behaviors (Fig. 2B) a two-way repeated measures ANOVA was utilized with main effects of time, (5 min sampling windows over 45 min) treatment (17β-estradiol vs. vehicle), and time-by-treatment interaction. *Post-*hoc group comparisons were evaluated using a Fisher’s LSD test. Regression analyses were utilized to examine putative causal relationships between variables. For the in vitro data presented in Fig. 6, a one-way ANOVA (with *post*-hoc analysis) was utilized to determine differences between treatment groups. Data are presented as means ± SD (unless otherwise noted) and p < 0.05 was always considered statistically significant. Statistically significant outliers were determined (Grubbs’ method with an Alpha = 0.05) and excluded from analyses, with corresponding analyses of complete data sets provided in the Supplementary Information. Data were reported missing when a sample could not reliably be obtained and are appropriately acknowledged in each figure legend. Exclusions for behavioral analyses were based on visual identification of compromised (e.g., acute minor injury) or abnormal (e.g., excessive jumping or attempting to exit the apparatus) locomotor behavior presumed unrelated to infection and/or treatment. Across 4 cohorts, there were 3 such cases in the vehicle group and 3 cases in the 17β-estradiol group. These individuals were excluded from all behavioral analyses.

### Ethics approval and consent to participate

All the experiments were performed according to the National Institutes of Health guidelines and regulations. The Institutional Animal Care and Use Committee of Cedars-Sinai Medical Center (protocol #7914) approved all the experimental procedures. All animals were kept under regular barrier conditions at room temperature with exposure to light for 12 h and dark for 12 h. Food and water were provided ad libitum. All efforts were made to ameliorate the suffering of animals. Deeply anesthetized mice were euthanized by perfusion followed by a physical method for tissue collection.

## Results

### Oophorectomy (OVX) and UTI-mediated changes in CC3

Figure 1A depicts the design of a preliminary experiment examining the effects of oophorectomy (OVX), UTI, and OVX + UTI on levels of cortical and hippocampal CC3. Figure 1B shows representative frontal cortex and hippocampal regions of interest (ROI) and Fig. 1C depicts representative staining in all three groups. Following UTI induction, compared to non-OVX mice, OVX mice demonstrated significantly increased levels of frontal cortex and hippocampal CC3, a neuronal injury marker that we previously demonstrated to correlate with delirium-like behaviors in mice with UTI^13^ (Fig. 1D,E; p < 0.001) and both measures were predictive of one another (R^2^ = 0.8635, p < 0.001; Fig. 1F). Mice oophorectomized, but without UTI induction, had less cortical and hippocampal CC3 than groups with UTI (both sham and OVX) indicating the potential for the combined effects of systemic alterations on CC3 levels (Fig. 1D,E; p < 0.05).

### 17β-estradiol significantly ameliorates UTI-induced delirium-like behavior

Figure 2A delineates schematics of the primary experimental design, treatment schedule of 17β-estradiol, and behavioral test apparatuses. Mice were oophorectomized, behavioral tests (*pre*) performed, and animals were then inoculated with *E. coli* to initiate UTIs. Following UTI induction, mice were treated for 3 consecutive days with 17β-estradiol or a vehicle, behavioral (*post*) tests performed and then sacrificed for tissue collection. Open field and elevated plus maze behavioral tests were performed prior to UTI induction (*pre*) and following treatment dose 2 (*post*). To prevent habituation to the apparatus and lack of motivation in subsequent exposure; novel object recognition and Y-maze behavioral tests were only carried out *post* UTI induction and treatment.

In the open field test, following oophorectomy, but prior to UTI induction and 17β-estradiol treatment (OVX + Pre-UTI), two-way repeated measures ANOVAs revealed a significant main effect of time, showing a decreased distance travelled (F_3,110_ = 22.2; p < 0.0001) and increased time spent immobile (F_5,171_ = 14.8; p < 0.0001) over the course of the 45-min test, typical of habituation to the environment (Fig. 2B). Prior to UTI induction there were no significant effects of treatment or time-by-treatment interactions. When retested in the open field test following UTI (OVX + Post-UTI), repeated measures ANOVAs indicated a significant time-by-treatment interaction for distance traveled (F_24,496_ = 1.9; p = 0.0052) and time spent immobile (F_24,448_ = 3.0; p < 0.0001), indicating vehicle-treated mice did not show habituation to the chamber (constant distance travelled and time spent immobile over 45-min session), likely reflecting psychomotor agitation. Treatment with 17β-estradiol prevented psychomotor agitation and mice generally showed the same patterns of activity/immobility as OVX + Pre-UTI. Results of post-hoc tests, comparing treatment effects (vehicle vs. 17β-estradiol) at individual time points revealed significant differences at multiple 5-min intervals (Fig. 2B). Additionally, mice (OVX + Post-UTI) treated with 17β-estradiol spent less time in the periphery of the open field (Fig. 2C, p < 0.05), indicating less anxiety-like behaviors. These findings were corroborated in the elevated plus maze where animals treated with 17β-estradiol spent more time in the open arms of the apparatus (during the first 6 min of a 10 min test) compared to vehicle treated controls (Fig. 2D, p < 0.05). Additionally, mice treated with 17β-estradiol defecated significantly less compared to vehicle treated controls during the open field test (Fig. S1, p < 0.05), potentially indicating less emotionality or anxiety^34^. Importantly, any behavioral differences between groups were not attributable to differences in overall locomotor activity, as there were no statistically significant differences in total distance traveled or average speed between 17β-estradiol- and vehicle-treated animals prior to or following UTIs (Fig. S2, p > 0.05).

For the novel object recognition test, mice treated with 17β-estradiol spent significantly more time oriented towards the novel object compared to vehicle treated controls (Fig. 2E, p < 0.05), indicating less impaired attention/short-term memory performance. There was no difference between groups in amount of time oriented toward the known object (results not shown). Finally, mice treated with 17β-estradiol demonstrated significantly increased percent spontaneous alternations in the Y-maze compared to vehicle treated controls (Fig. 2F, p < 0.01), further supporting improved short-term memory performance.

Importantly, UTI severity was not significantly different between sham and OVX mice in the initial experiment or between 17β-estradiol- and vehicle-treated animals in the primary experiment (Fig. S3, p > 0.05), indicating differential infection is not underlying any structural or functional effect. Additionally, bacterial burden did not explain a significant amount of the variability in the systemic inflammatory response as measured by plasma IL-6 (Fig. S3c).

### 17β-estradiol decreases UTI-induced CC3 in the frontal cortex and hippocampus

**Figure 3:**
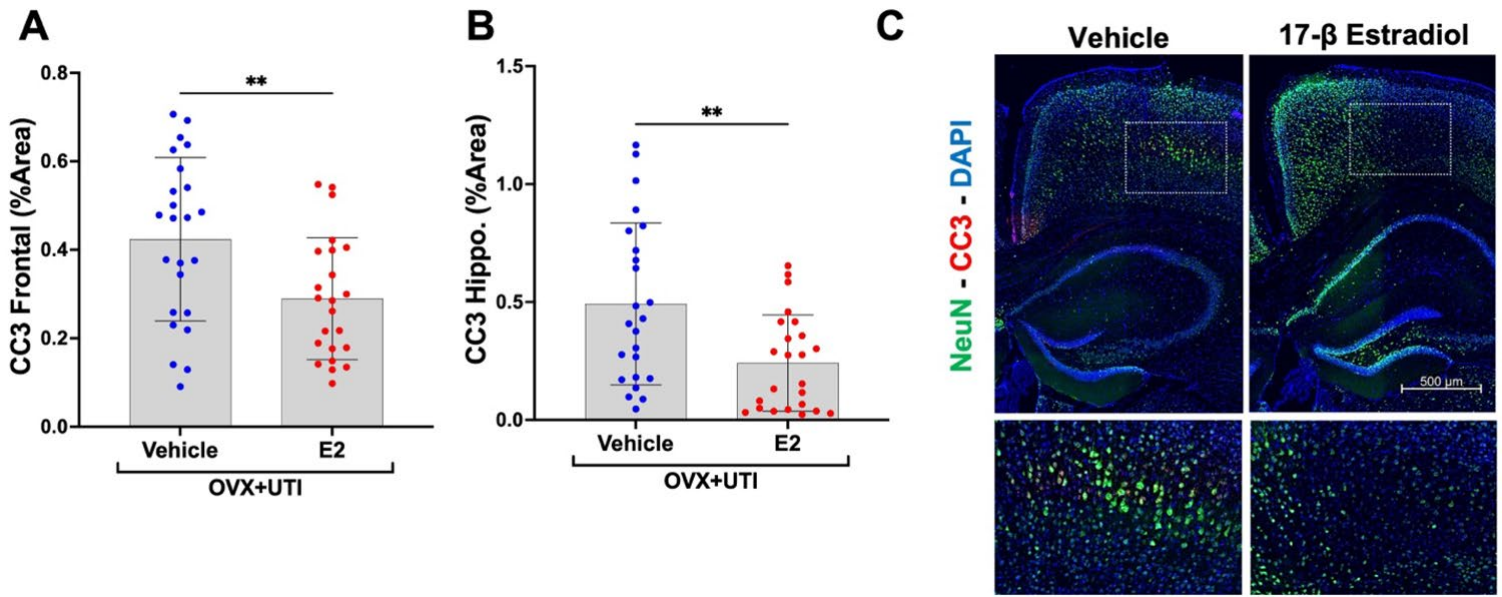
17β-estradiol (E2) decreases UTI-induced cleaved caspase-3 (CC3) in the frontal cortex and hippocampus. (**A**,**B**) Among oophorectomized mice with UTIs (OVX + UTI), those treated with E2 (n = 24) show a statistically significant reduction in CC3 in both the frontal cortex and hippocampus as compared to vehicle (sesame oil) treated (n = 24). (**C**) Representative staining of CC3 and neuronal nuclear protein (NeuN) in both vehicle and E2 treated mice. For (**A**), a single statistical outlier was identified and removed from the E2 group. Exclusion of the data point yielded p = 0.0072 (t = 2.814, df = 45), while inclusion resulted in p = 0.0352 (t = 2.170, df = 46) (see Fig. S7a) *p < 0.05, **p < 0.01.

**Figure 4:**
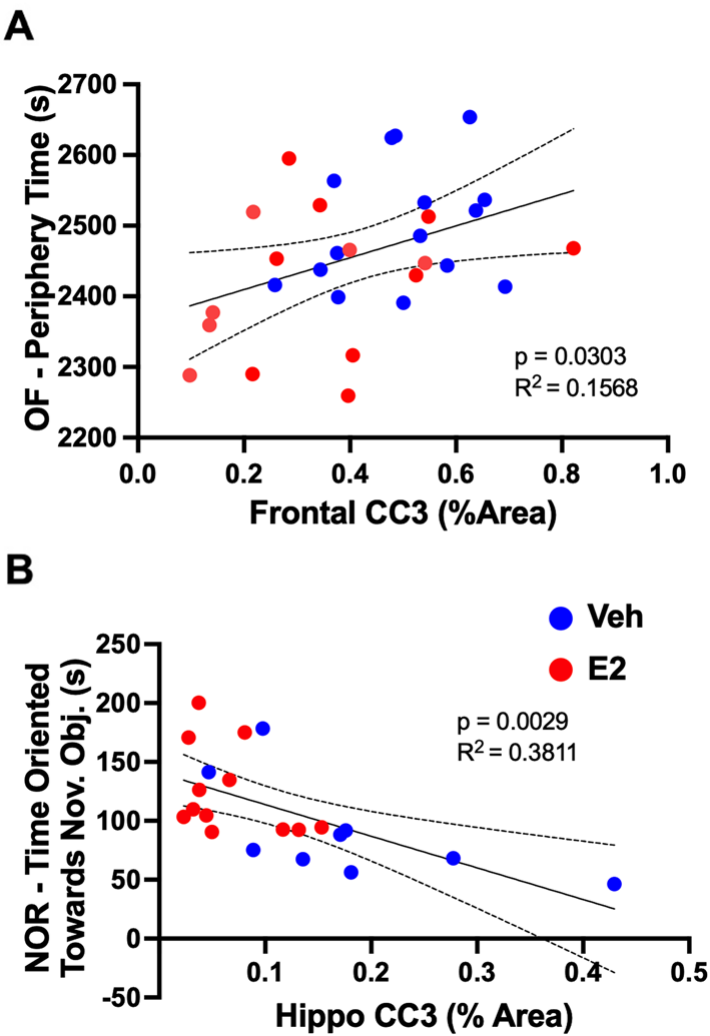
Cleaved caspase-3 (CC3) variability explains delirium-like behaviors. (**A**) Regression analysis across groups (n = 21–30) demonstrates a statistically significant positive relationship between frontal cortex cleaved caspase-3 (CC3) and time spent in the periphery during an open field test (OF). (**B**) Regression analysis reveals a significant negative relationship between hippocampal CC3 and time oriented towards a novel object during a novel object recognition test (NOR). Veh = vehicle treated; E2 = 17β-estradiol treated. Dotted lines represent 95% confidence intervals.

Figure 3 displays levels and representative staining of CC3, a neuronal injury marker. Among OVX mice with UTIs (OVX + UTI), administration of 17β-estradiol significantly reduced CC3 levels in the frontal cortex and hippocampus compared to vehicle-treated mice (Fig. 3A–C; p < 0.05). Notably, improvements in frontal cortex and hippocampal CC3 levels were associated with corresponding ameliorations in acute UTI-mediated delirium-like behavioral changes across the multiple tests, supporting the role of CC3 as a neuronal injury marker of delirium-like behavior. At the level of the individual, CC3 abundance explained a statistically significant amount of variation in some, but not all, behaviors associated with increased anxiety and memory deficits (Figs. 4 and S4). These significant relationships were observed despite the study not being specifically statistically powered for regression analyses, but instead powered to detect group differences. With adequate statistical power we believe we may have observed additional significant relationships, especially for those depicted in Fig. S4a–d. However, we also acknowledge that by only utilizing CC3 as a single neuronal injury marker we are almost certainly not capturing the entirety of the underlying pathophysiological mechanisms responsible for the UTI-induced delirium-like behavior.

### 17β-estradiol attenuates IL-6 concentration in the plasma and frontal cortex

**Figure 5:**
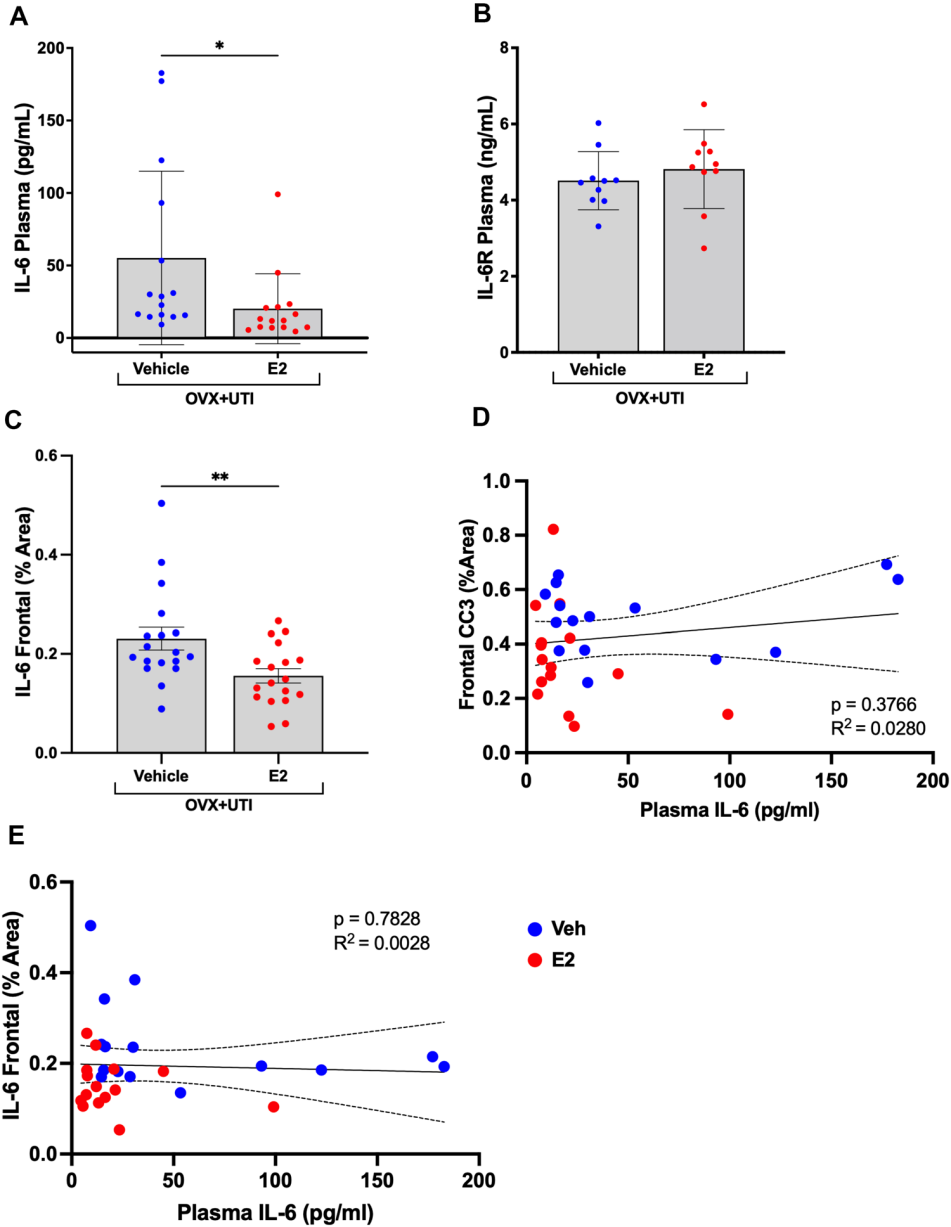
17β-estradiol (E2) attenuates plasma and frontal cortex concentrations of IL-6 but not soluble IL-6 receptor alpha (sIL-6R). (**A**–**C**) Among ovariectomized and UTI inoculated mice (OVX + UTI), individuals treated with E2 (7 µg) have significantly lower IL-6 plasma concentrations in the plasma and frontal cortex with no significant change in sIL-6R concentrations as measured via ELISA. Plasma samples (n = 15) could reliably be obtained from all but 3 vehicle and 2 E2 animals. For (**B**), plasma IL-6R was measured in a randomly chosen subset of animals (n = 10/group) to confirm differences in delirium-like phenotypes were not driven by receptor density. For (**A**), a single statistical outlier was identified and removed from the E2 group – the abnormally high value may have resulted from hemolysis of the sample. Exclusion of the data point yielded p = 0.0495 (t = 2.102, df = 18.45), while inclusion resulted in p = 0.1654 (t = 1.430, df = 24.66) (Fig. S7). (**D**,**E**) Regression analyses of plasma IL-6 (pg/ml) on cleaved caspase-3 (CC3) and IL-6 levels in the frontal cortex. Dotted lines represent 95% confidence intervals. Group size was n = 10–18. Data are expressed as mean ± SD. *p < 0.05, **p < 0.01.

We previously demonstrated that female mice with UTIs have significantly higher plasma IL-6, increased delirium-like behaviors, and elevated neuronal CC3 compared to non-UTI control mice^13^. We also previously demonstrated that plasma IL-6 was positively related to and explained a significant amount of the variation in CC3 levels^13^. Here, following oophorectomy and UTI induction, mice treated with 17β-estradiol had significantly lower IL-6 plasma concentrations (Fig. 5A, p < 0.05) with no significant change in sIL-6R concentrations (Fig. 5B, p > 0.05) in plasma collected 3 days following UTI induction. Plasma IL-6R was measured in a randomly chosen subset of animals (n = 10/group) to confirm differences in delirium-like phenotypes were not driven by receptor density. These findings were associated with decreased expression of IL-6 in the frontal cortex of mice treated with 17β-estradiol (Fig. 5C, p < 0.01). Finally, regression analyses indicated plasma IL-6 explained only 2.8% of the variation in frontal CC3 levels across groups (Fig. 5D, R^2^ = 0.0280, p = 0.3766) and only 0.28% of cortical IL-6 levels (Fig. 5E, R^2^ = 0.0028, p = 0.7828). As estrogen supports urothelial defense mechanisms^35^, we performed H&E staining of a subset of randomly chosen animals and observed no difference in inflammatory cell infiltrates between E2 and vehicle treated animals (Fig. S5). Overall, these findings are consistent with decreased activation of the IL-6 *trans*-signaling pathway via a reduction in plasma and cortical IL-6, which we have previously hypothesized contributes to neuronal injury/neurodegeneration and ultimately delirium-like behaviors.

### 17β-estradiol reverses UTI-mediated neuronal changes

**Figure 6:**
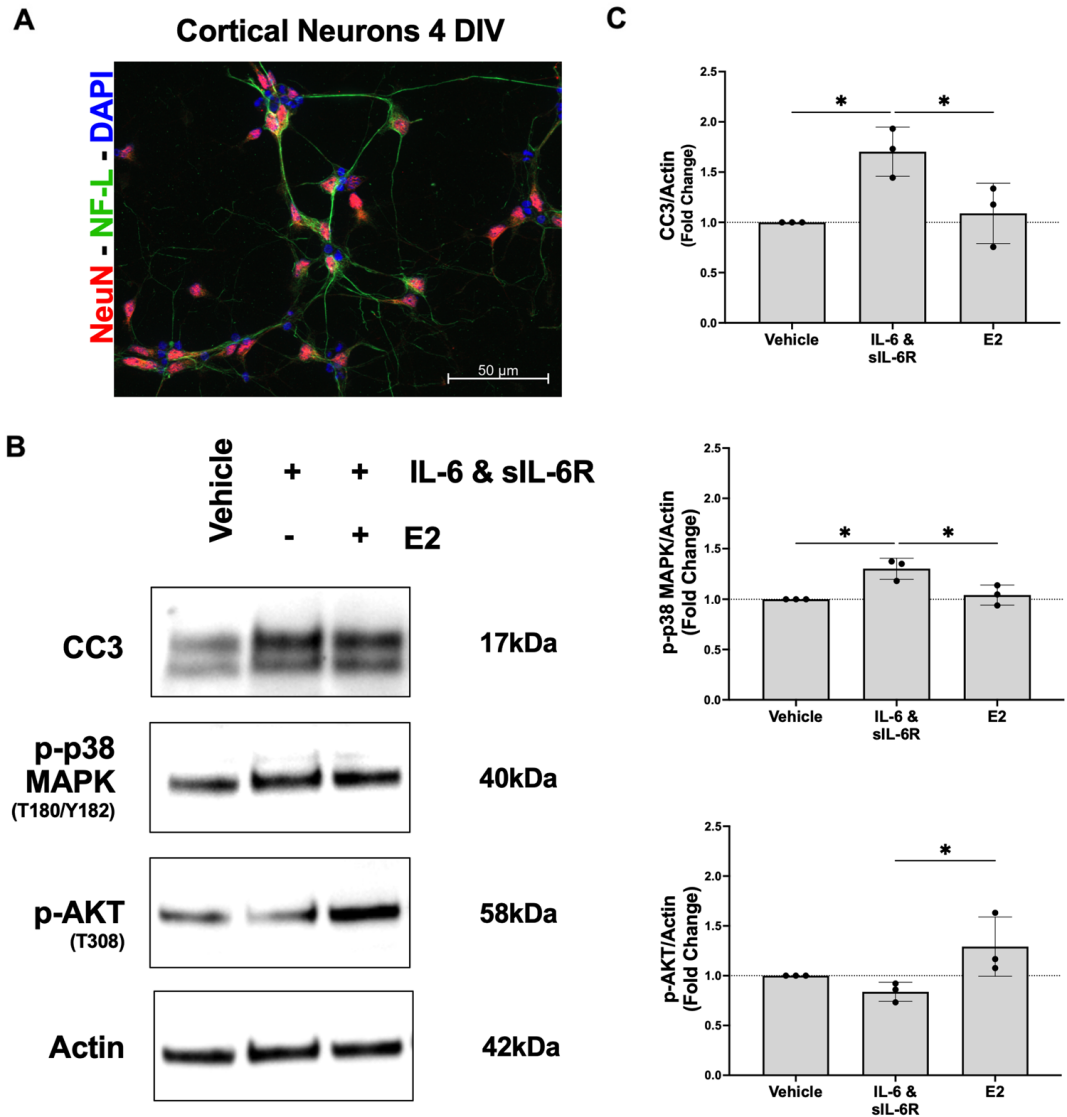
17β-estradiol (E2) reverses UTI-mediated neuronal changes. (**A**) Representative staining of primary rat cortical neurons 4 days in vitro (DIV) showing expression of two neuron-specific markers, neuronal nuclear protein (NeuN, red) and neurofilament-light chain (NF-L, green). (**B**) At 7 DIV, cortical neurons were treated for 1 h with either vehicle (growth medium), a combination of IL-6/sIL-6R, or combination of IL-6/sIL-6R plus E2. Representative western blot images of relevant signaling molecules that contribute to apoptosis, neurogenesis, and synaptic activity. Full unedited gel images are provided in supplementary information (Fig. S6). (**C**) Summary plot for fold changes relative to vehicle are presented for the indicated signaling molecules. Neurons treated with IL-6/sIL-6R had statistically significantly more cleaved caspase-3 (CC3) and p-p38 MAP kinase (MAPK) compared to the vehicle and those treated with combination of IL-6/sIL-6R plus E2 (n = 3). There was no significant difference in CC3 or p-p38 MAPK levels between the vehicle and neurons treated with IL-6/sIL-6R plus E2 (n = 3). Neurons treated with treated with combination of IL-6/sIL-6R plus E2 had significantly more p-AKT than the vehicle or those treated with only IL-6/sIL-6R (n = 3). IL-6 was always administered with sIL-6R since neurons do not express the IL-6 receptor. As an additional control we quantified CC3 in the presence of IL-6 alone and found no difference compared to vehicle (Fig. S8). Data are expressed as mean ± SD. *p < 0.05.

We used an in vitro model to directly examine the toxic effects of IL-6/sIL-6R, and the potential protective effects of 17β-estradiol, in rat cortical neurons. Figure 6A represents staining of primary rat cortical neurons 4 days in vitro (DIV) showing expression of two neuron-specific markers, neuronal nuclear protein (NeuN, red) and neurofilament-light chain (NF-L, green). At 7 DIV, cortical neurons treated with a combination of IL-6 /sIL-6R showed statistically significant increases in signaling molecules associated with neuronal dysfunction (CC3, p-p38MAPK) and significant decreases in those associated with neuronal survival (p-AKT) (Fig. 6B,C). Full unedited gel images are provided in supplementary information (Fig. S6). Supplementation of 17β-estradiol in conjunction with IL-6/sIL-6R largely reverses changes induced by IL-6/sIL-6R alone. These in vitro results provide support for the *trans*-signaling pathway hypothesis contributing to IL-6 mediated neuronal injury and 17β-estradiol ameliorating the effects.

## Discussion

In this study, we demonstrate a key protective role for 17β-estradiol in acute UTI-induced structural and functional delirium-like phenotypes in a mouse model. Further, we demonstrate that 17β-estradiol may protect the brain via pleiotropic mechanisms including ameliorating IL-6 production and directly blocking the toxic effects IL6/IL6R in neurons. These pre-clinical results call for future clinical investigations using acute 17β-estradiol therapy to ameliorate acute delirium-like states following UTI in postmenopausal women.

While this study is the first to demonstrate a protective role for 17β-estradiol in UTI-mediated acute delirium-like phenotypes, prior animal studies have demonstrated the hormone’s protective effects against cognitive decline^36–39^. In one study, 17β-estradiol administration to oophorectomized wild-type mice enhanced hippocampus-dependent memory and increased estrogen receptor activation in the hippocampus and cortex^36^, while another found that 17β-estradiol administration to aged female or male mice improved performance in frontal cortex and hippocampus-mediated tasks^37^. In a model of experimental autoimmune encephalomyelitis, 17β-estradiol promoted anti-inflammatory effects and improved disease severity^40,41^. Although research on estrogen replacement in humans has yielded mixed results, additional support for the role of estrogen in mediating cognitive function comes from clinical studies that show that younger women who lose endogenous estradiol from early oophorectomy appear to be at high risk for impairments in frontal cortex and hippocampus mediated cognitive function^42–47^. The findings from the current study add to this existing body of work to demonstrate a role for 17β-estradiol in ameliorating acute delirium-like phenotypes.

**Figure 7:**
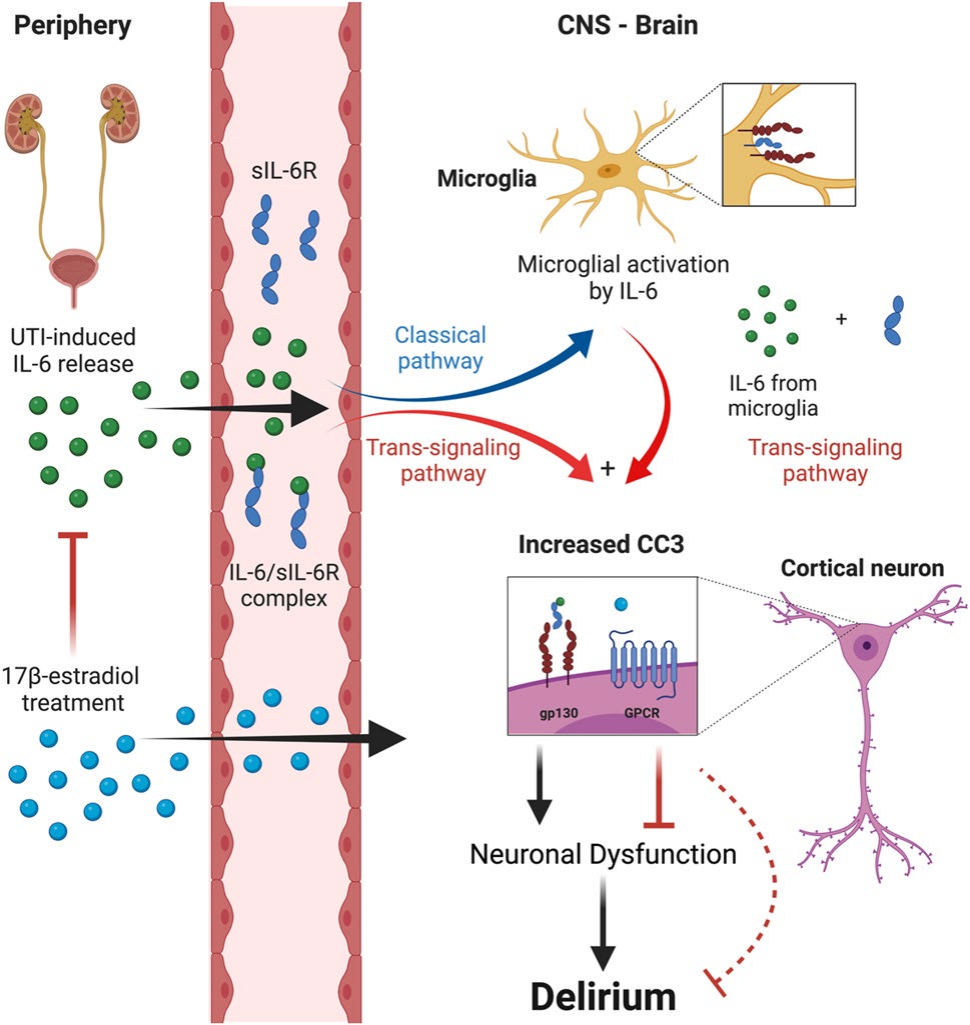
Hypothesized schematic of the IL-6 *trans*-signaling pathway and protective effects of 17β-estradiol. UTI-induced increases in IL-6 and soluble IL-6R (sIL-6R) complex in the periphery and directly induce neuronal dysfunction (i.e., increased frontal cortex and hippocampal CC3) via the gp130 transmembrane protein. 17β-estradiol reduces systemic and central inflammation and provides protective effects via reduction in neuronal CC3 mediated through neuronal G-protein estrogen receptor. Figure is adapted from Hogg et al.^55^. Created with BioRender.com.

Our findings suggest that the neuroprotective effects of 17β-estradiol occur via peripheral modulation of IL-6 as indicated by reduced plasma levels and decreased expression of IL-6 in the frontal cortex and hippocampus. Although beyond the scope of this study, these effects are likely to be mediated via immune cells, such as macrophages, in the peripheral circulation resulting in lower IL-6 concentrations in the blood^22^. The lowering of peripheral IL-6 is believed to mitigate neuronal injury via reduced activation of the IL-6 *trans*-signaling pathway that has been described to induce neurological injury in diverse neurodegenerative conditions^48,49^. However, plasma and frontal IL-6 values are not significantly related (Fig. 5E). We believe this may be a timing issue as our current hypothesis is that infection causes systemic inflammation (i.e., increases in IL-6), followed by brain inflammation and subsequent neuronal dysfunction and behavioral impairment (Fig. 7). Measuring both plasma and frontal IL-6 levels at the same time point (3 days *post* infection) may lead to the false impression that the levels are not predictive of each other—as plasma IL-6 levels may begin to fall as cortical levels are stable or increasing. Future experiments should examine the spatiotemporal expression and origins of IL-6 after UTI and how systemic increases correspond to brain levels across the entirety of infection. Our in vitro data also suggest that estradiol may directly exert protective effects on neurons, suggesting that pleiotropic mechanisms likely underlie estradiol-mediated neuroprotection in UTI. Currently, it is common clinical practice to use antibiotics to ameliorate UTI-induced delirium even though there are no randomized controlled trials that indicate this practice is effective. This equipoise is reflected in current clinical practice guidelines that do not support treatment of bacteriuria with delirium as the isolated symptom^50^. Thus, future studies are needed to determine if immunomodulation more effectively ameliorates UTI-induced delirium compared to antibiotics.

Our study has several important strengths including the use of a novel animal model of UTI-induced delirium to demonstrate 17β-estradiol’s beneficial effects on both structural, i.e., CC3, and multiple behavioral changes that closely resemble the clinical phenotype of delirium. Further, 17β-estradiol is a physiological hormone and existing pharmacotherapeutic agent that can be readily translated to clinical trials to treat delirium—the results of this research provide pre-clinical justification for using 17β-estradiol to address delirium due to UTI. Several limitations of this study warrant consideration. First, to isolate the variable of the postmenopausal period, we used middle-aged mice who may not represent the population at highest risk for developing delirium. However, delirium is not only a geriatric condition and is prevalent in all age ranges, including in pediatric populations^51^. Nevertheless, future studies should examine the interaction of older age and the postmenopausal period on delirium phenotypes. Second, although we showed beneficial effects of 17β-estradiol, we still do not know if the effect is mediated via classical estrogen receptors or alternative pathways such as the G-protein estrogen receptor. Given the rapid protective effects of estrogen in our studies, it is possible that non-genomic pathways, e.g., via G-protein estrogen receptor, are involved and future studies are needed to evaluate these potential molecular pathways. Although frontal/hippocampal CC3 accounted for a portion of the variability in delirium-like behaviors, it remains possible that additional cellular markers would further strengthen the relationship between the measured UTI-induced brain structural and functional changes. Thus, future studies, perhaps using proteomics, metabolomics, and transcriptomics are needed to identify novel cellular markers as robust correlates of the delirium-like behaviors. Additionally, future studies are also needed to evaluate the effect of direct CC3 inhibition on delirium-like phenotypes, as depicted in Fig. 7. Third, we used a relatively high volume of inoculum which has the potential to cause vesicoureteral reflux and pyelonephritis^52^. While we administered the inoculum as slowly as possible to minimize the risk of vesicoureteral reflux and pyelonephritis, it remains possible that pyelonephritis occurred in some animals^53,54^. Future experiments are needed to assess if severity of delirium-like phenotypes vary in the presence of cystitis or pyelonephritis. Finally, although we demonstrated two potential pathways by which 17β-estradiol may mitigate delirium, given the multitude of pleiotropic effects of the hormone, there could be other mechanisms, immune-mediated or otherwise, that are also involved.

In conclusion, in this paper, we used murine and in vitro models to demonstrate a novel role for 17β-estradiol in ameliorating UTI-induced acute delirium-like phenotypes. These results call for future clinical trials using 17β-estradiol to mitigate delirium due to UTI.

## Supplementary Information

Supplementary Information.

## Data Availability

The data sets used and/or analyzed during the current study are available from the corresponding author on reasonable request.

## Abbreviations

UTI: Urinary tract infection
IL-6: Interleukin-6
CC3: Cleaved caspase-3
OVX: Oophorectomized
*E. coli*: *Escherichia coli*
CFU: Colony forming units
ROI: Regions of interest
EPM: Elevated plus maze
NOR: Novel object recognition
ANOVA: Analysis of variance
DIV: Days in vitro
E2: 17β-Estradiol
OF: Open field
NeuN: Neuronal nuclear protein
NF-L: Neurofilament-light chain
MAPK: P-p38 MAP kinase
