# Supplementary material for: 17β-estradiol ameliorates delirium-like phenotypes in a murine model of urinary tract infection
Source: Sci Rep. 2022 Nov 15;12:19622. doi: 10.1038/s41598-022-24247-w (PMC9666646; doi:10.1038/s41598-022-24247-w)
Supplement: Supplementary file 1 — Supplementary Information. [file 41598_2022_24247_MOESM1_ESM.pdf]

Supplementary Information

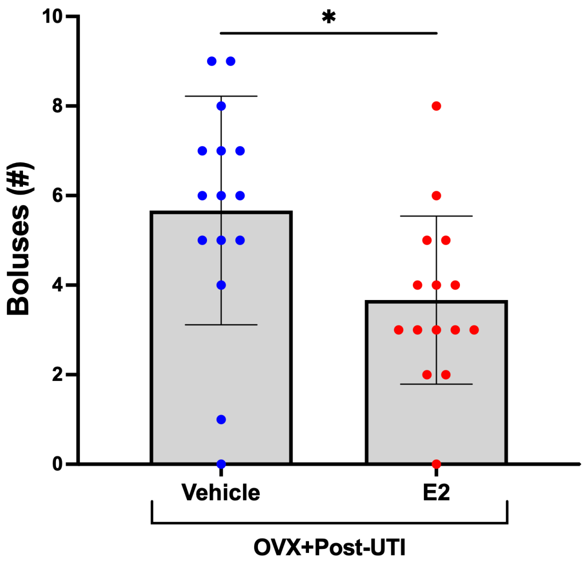

Figure S1. 17β-estradiol (E2) significantly reduces UTI-induced emotionality. Among oophorectomized and UTI inoculated mice (OVX+UTI), animals treated with E2 defecated statistically significantly less during an open field test compared to vehicle treated (sesame oil) controls (n = 15 / group). Data are expressed as mean ± SD. *p < 0.05.

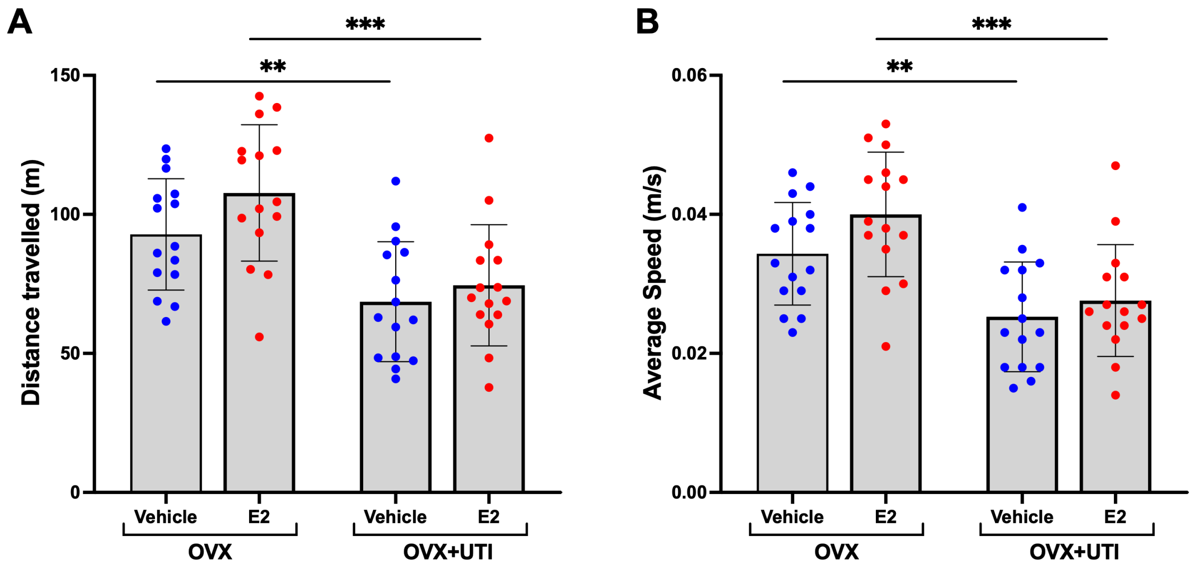

Figure S2. 17β-estradiol (E2) does not significantly alter general locomotor activity behavior. (**a, b**) Following oophorectomy, but prior to UTI induction and E2 treatment (OVX), there were no statistically significant differences between groups (n = 15 / group) in distance traveled (m) or average speed (m/s) in an open field test. Among oophorectomized and UTI inoculated mice (OVX+UTI), individuals treated with E2 did not significantly differ in distance traveled (m) or average speed (m/s) in an open field test compared to vehicle treated (sesame oil). All mice, regardless of treatment group, tended to be less active following UTI induction. Data are expressed as mean ± SD. $^{**}p < 0.01$, $^{***}p < 0.001$.

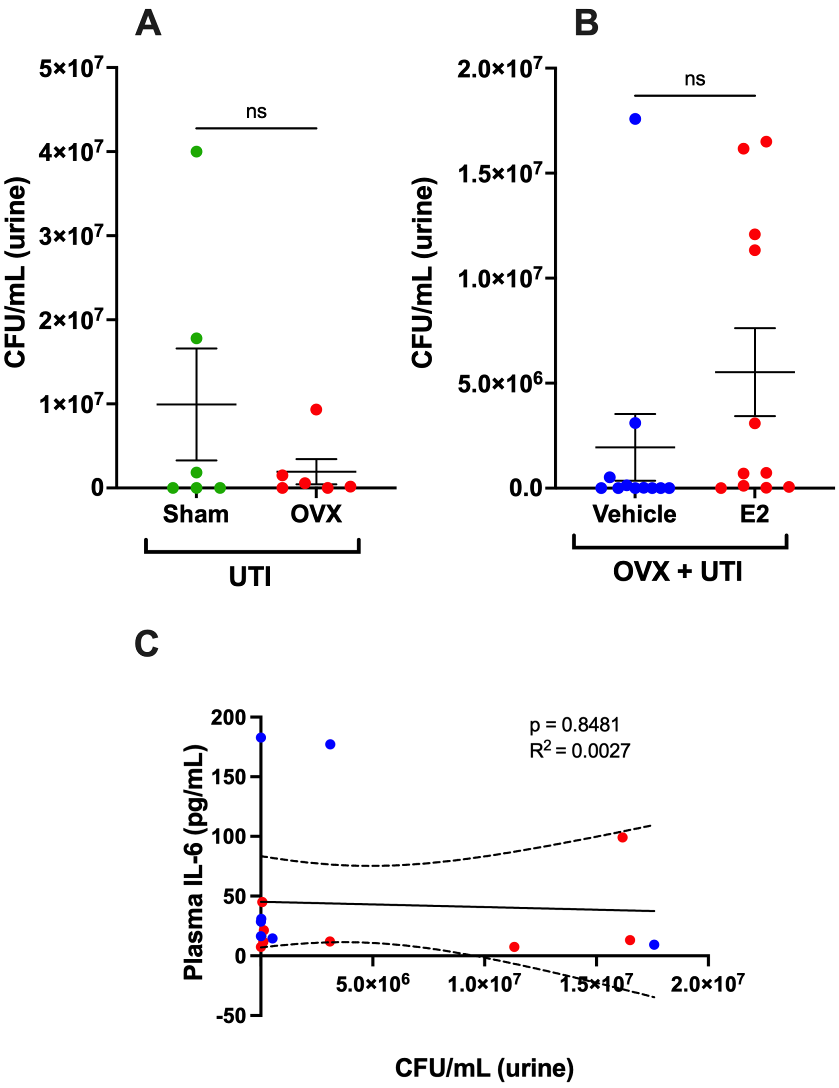

Figure S3. Transurethral inoculation of *E. coli* causes UTI. Bacterial enumeration was quantified 72 hrs. *post* inoculation. Animals for which urine could not be obtained in sufficient volume were excluded from this analysis. (**a**) In the initial experiment (Fig. 1), there was no significant difference in bacterial burden [as measured by colony forming unit (CFU)] between mice with UTI (sham; n = 6, mean = 9.9 x 10^6) and oophorectomized mice with UTI (OVX+UTI; n = 6, mean = 1.9 x 106) (p = 0.2675; t = 1.174, df = 10). (**b**) In the primary experiment (Figs. 2-5), oophorectomized mice with UTI (OVX+UTI) treated with 17β-estradiol (E2, n = 11, mean = 5.53 x 10^6) did not have statistically significantly different bacterial burden (CFU) compared to vehicle treated oophorectomized mice with UTI (sesame oil, n = 11, mean = 1.9 x 10^6) (p = 0.1878; t =

1.364, df = 20). (**c**) Regression analysis revealed that bacterial burden (CFU) 3 days *post* infection did not explain a significant amount of the variability in plasma IL-6 levels. Data are expressed as mean ± SEM. Dotted lines represent 95 % confidence intervals.

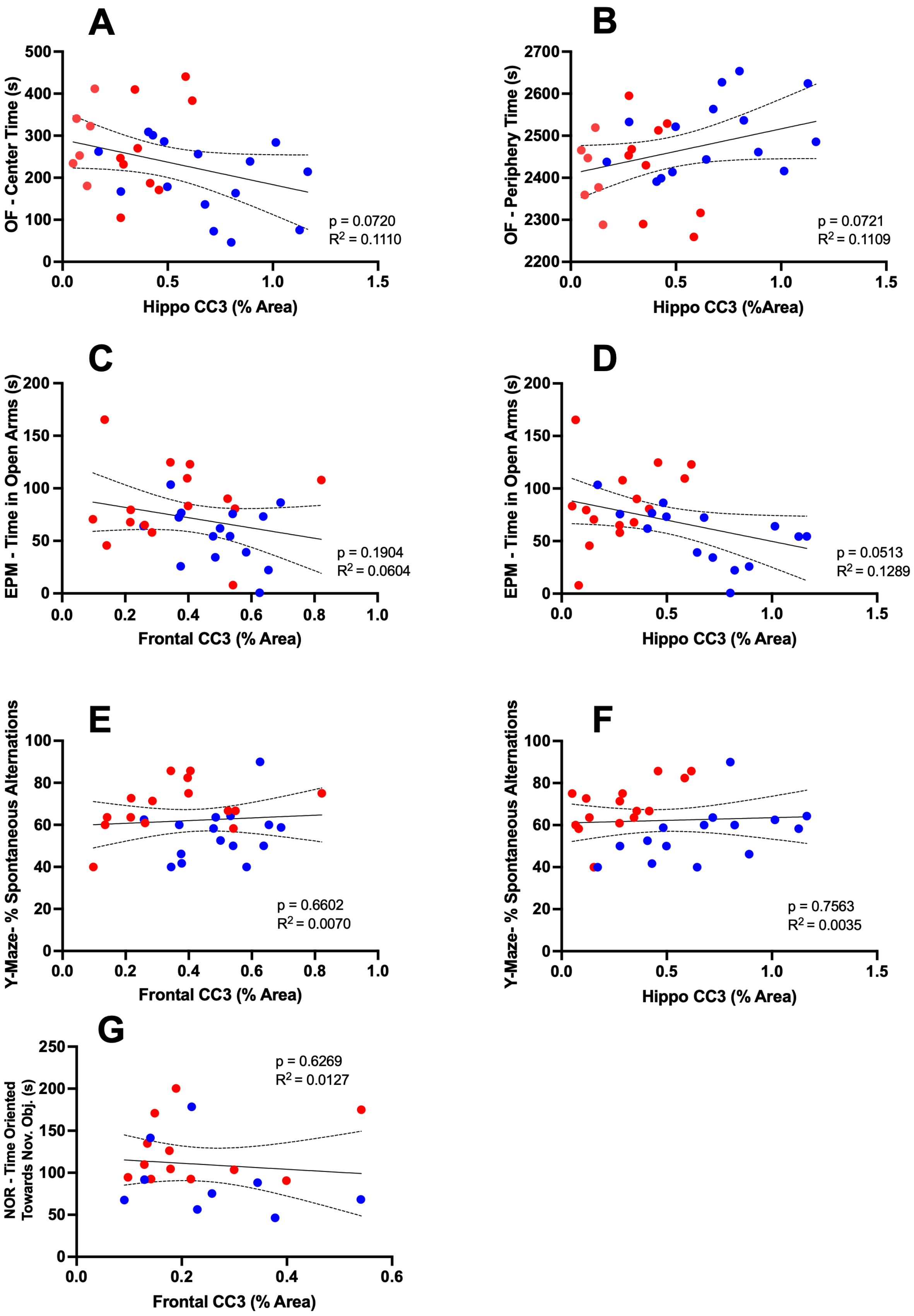
A
OF - Center Time (s)
Hippo CC3 (% Area)
p = 0.0720
R^2 = 0.1110
B
OF - Periphery Time (s)
Hippo CC3 (%Area)
p = 0.0721
R^2 = 0.1109
C
EPM - Time in Open Arms (s)
Frontal CC3 (% Area)
p = 0.1904
R^2 = 0.0604
D
EPM - Time in Open Arms (s)
Hippo CC3 (% Area)
p = 0.0513
R^2 = 0.1289
E
Y-Maze- % Spontaneous Alternations
Frontal CC3 (% Area)
p = 0.6602
R^2 = 0.0070
F
Y-Maze- % Spontaneous Alternations
Hippo CC3 (% Area)
p = 0.7563
R^2 = 0.0035
G
NOR - Time Oriented Towards Nov. Obj. (s)
Frontal CC3 (% Area)
p = 0.6269
R^2 = 0.0127

Figure S4. Associations of cleaved caspase-3 (CC3) with UTI-induced behavior. (**a-g**) Regression analyses across groups (n = 21-30) of relationships between frontal cortical and hippocampal CC3 with delirium-like behaviors. Behavioral tests utilized were open field (OF), elevated plus maze (EPM), novel object recognition test (NOR), and Y-maze. Veh = vehicle treated; E2 = 17β-estradiol treated. Dotted lines represent 95 % confidence intervals.

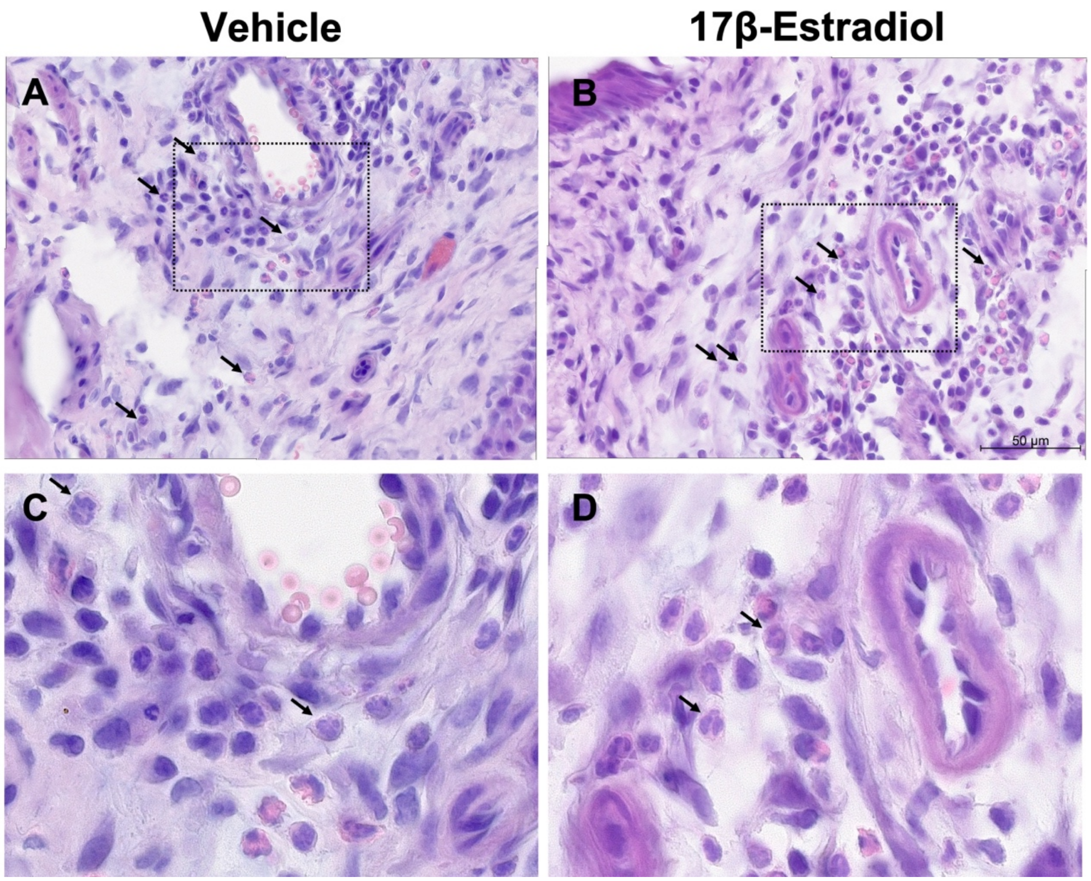

Figure S5. We performed H&E staining of a subset of randomly chosen animals and observed no difference in inflammatory cell infiltrates in bladder tissue (mucosal layer) between E2 and vehicle treated animals. Representative images are displayed. (**a**) Both vehicle and 17β-estradiol treated mice show evidence of inflammatory cell infiltrates (examples indicated by arrows). Low (**a**, **b**) and high (**c**, **d**) magnification of representative micrographs of bladder sections (8μm).

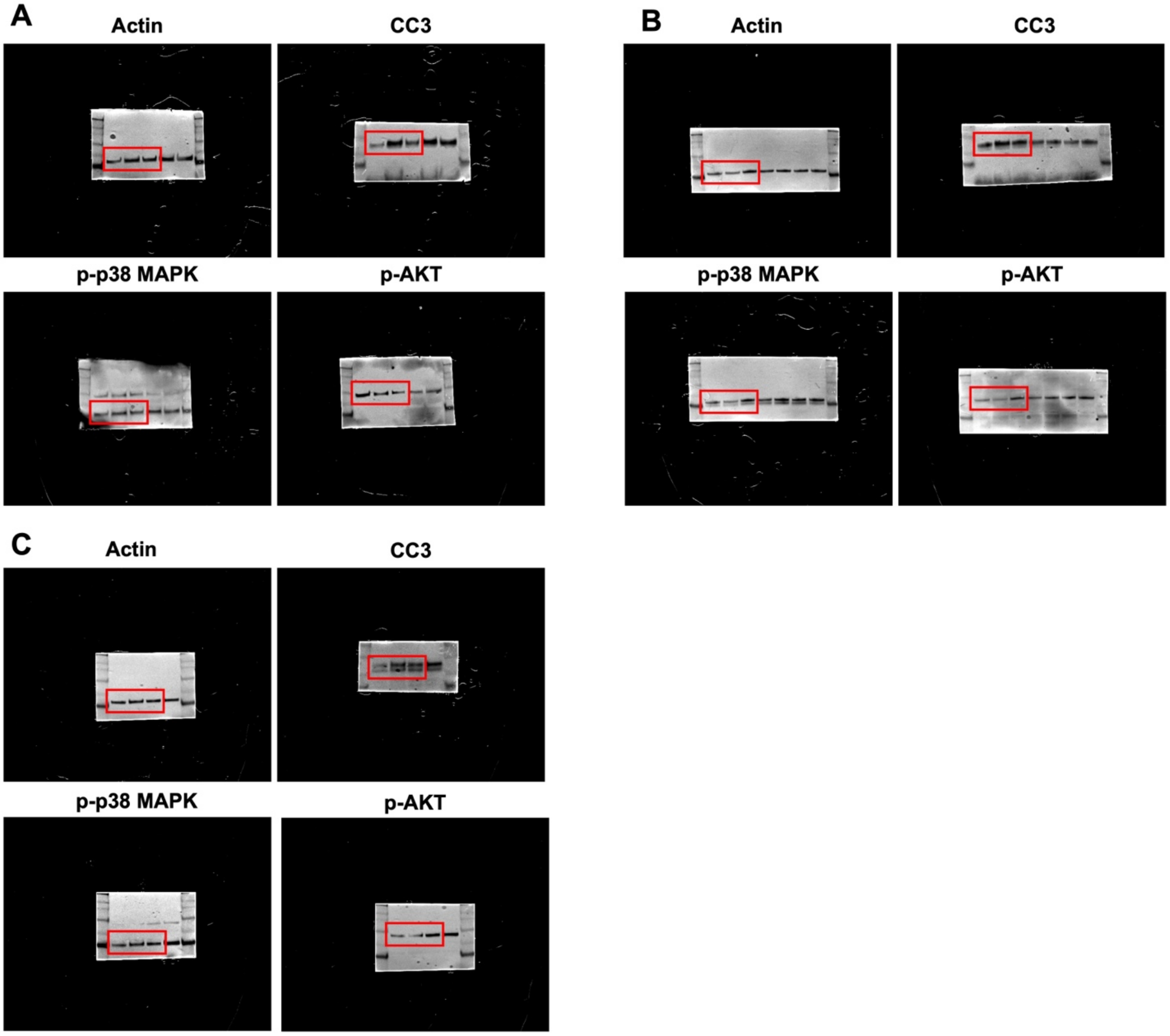

Figure S6. Full unedited gels corresponding to Figure 6b, c. (**a-c**) represent the 3 replicate experiments – with the replicate depicted in (**a**) shown in Fig. 6b. For each molecule, we have listed the utilized antibody in Table S1.

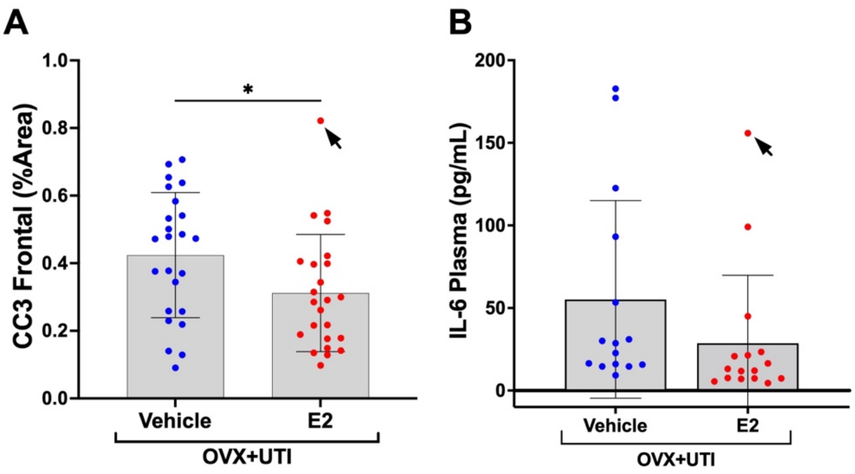

Figure S7. Data corresponding to Fig. 3a and 5a in the main text. (**a**) For CC3, a single statistical outlier was identified (indicated by arow) using Grubbs' method with an Alpha = 0.05. Exclusion of the data point yielded p = 0.0072 (t = 2.814, df = 45), while inclusion (depicted here) resulted in p = 0.0352 (t = 2.170, df = 46). For (**b**) plasma IL-6, a single statistical outlier was identified (indicated by arow) using Grubbs' method with an Alpha = 0.05. We believe the abnormally high value may have resulted from hemolysis of the sample. This single data point was removed, and an analysis yielded a p-value of 0.0495 (t = 2.102, df = 18.45). Here we present the figure with the data point included where the analysis resulted in p = 0.1654 (t = 1.430, df = 24.66). Data are expressed as mean ± SD.

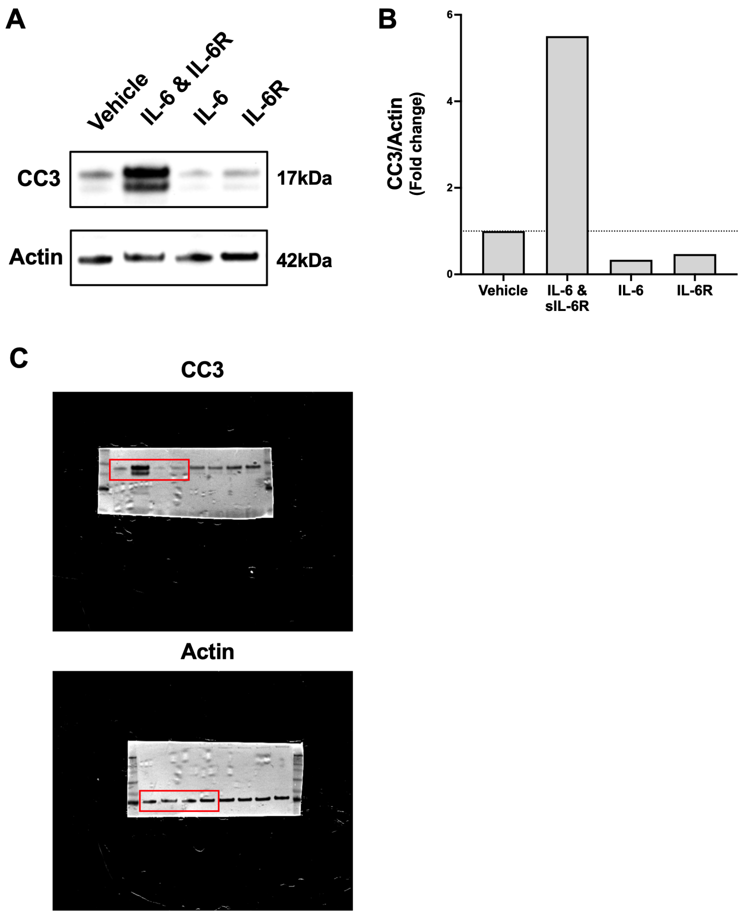

Figure S8. In Fig. 6, IL-6 was always administered with sIL-6R since neurons do not express the IL-6 receptor. As an additional control we administered IL-6 alone and quantified cleaved caspase-3 (CC3) in a single experiment (shown here). In the presence of IL-6 alone there was no statistically significant difference compared to vehicle. The edited (**a**) and unedited (**c**) blots are provided. For (**b**), vehicle (growth medium); IL-6 and sIL-6R (10 ng/mL and 100ng/mL, respectively); IL-6 (10 ng/mL), IL-6R (100 ng/mL).

Table S1. Western blot primary antibodies.

Antigen	Dilution	Vendor	Catalog #
Actin-HRP	WB=50,000	Life Tech.	MA5-15739-HRP
Cleaved Caspase-3	IF=800/WB=600	Cell Sig.	9664
Neurofilament-Light	IF=1000	Life Tech.	PA1-10000
NeuN	IF=600	Life Tech.	MA5-33103
p-AKT (Thr308)	WB=800	Cell Sig.	13038
p-P38 MAPK (Thr180/Tyr182)	WB=800	Cell Sig.	4511
